# A review of spatial capture–recapture: Ecological insights, limitations, and prospects

**DOI:** 10.1002/ece3.8468

**Published:** 2021-12-21

**Authors:** Mahdieh Tourani

**Affiliations:** ^1^ Faculty of Environmental Sciences and Natural Resource Management Norwegian University of Life Sciences Ås Norway

**Keywords:** capture‐recapture, hierarchical models, ecological modelling, population dynamics, imperfect detection, wildlife monitoring

## Abstract

First described by Efford (2004), spatial capture–recapture (SCR) has become a popular tool in ecology. Like traditional capture–recapture, SCR methods account for imperfect detection when estimating ecological parameters. In addition, SCR methods use the information inherent in the spatial configuration of individual detections, thereby allowing spatially explicit estimation of population parameters, such as abundance, survival, and recruitment. Paired with advances in noninvasive survey methods, SCR has been applied to a wide range of species across different habitats, allowing for population‐ and landscape‐level inferences with direct consequences for conservation and management. I conduct a literature review of SCR studies published since the first description of the method and provide an overview of their scope in terms of the ecological questions answered with this tool, taxonomic groups targeted, geography, spatio‐temporal extent of analyses, and data collection methods. In addition, I review approaches for analytical implementation and provide an overview of parameters targeted by SCR studies and conclude with current limitations and future directions in SCR methods.

## Graphical abstract

By conducting a literature review, I have highlighted realized applications of spatial capture–recapture, an analytical framework to reveal complex ecological state dynamics that are inherently difficult to observe.

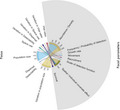

## 1 INTRODUCTION

Spatial heterogeneity in the environment shapes life‐history traits across individuals, scaling up to populations through coevolutionary processes (Levin, 1992). At the same time, populations and their impacts are manifested across the space they occupy. Many of the pressing problems faced by wildlife conservation and management revolve around species distribution and abundance, where these patterns and the underlying processes (e.g., vital rates) vary across time and space and can be associated with heterogeneity in the environment (Scheiner & Willig, 2008). As a result, quantifying variations in space, not only time, is pivotal for understanding ecological systems.

Making inferences about spatial variation in demographic processes advances our understanding of natural phenomena in a changing world. However, the ability to study populations at biologically meaningful extents has been hampered by available methods (Chandler et al., 2018). Both the interpretation and the use of information required to address applied challenges are scale‐dependent. The study of population dynamics benefits from fine‐scale, spatio‐temporal data to capture individual patterns and analytical methods to make inferences at the population level (Dunning et al., 1995; Royle et al., 2018). Novel sampling methods, such as noninvasive DNA sampling and camera trapping, have substantially increased the number of studies that collect data with a grain and extent suitable for addressing long‐standing questions related to population dynamics. However, there remain challenges to draw ecological inference from the resulting data.

One of the primary challenges in the study of populations or communities arises from the failure to detect all individuals; thus, inferences must be scaled up to the population based on a limited sample of questionable representativeness (Gimenez et al., 2008; Kellner & Swihart, 2014). Individuals exposed to sampling may remain undetected or vary in detection throughout the survey for different reasons; for example, because of variation in individual responses to sampling. Failure to account for imperfect detection may bias estimates of parameters of interest, leading to erroneous ecological inferences. As a solution, analytical methods that account for imperfect detection are now common in ecological studies (Kellner & Swihart, 2014; Kéry & Royle, 2016).

For decades, ecologists have relied on capture–recapture methods as a standard sampling and analytical framework to infer about population size and the underlying processes, such as survival and recruitment (Seber, 1986). Capture–recapture accounts for imperfect detection through repeated sampling and modeling the observation process, while the demographic component of the model concerns the ecological processes. Capture–recapture methods have not only deepened our understanding of demography in wild populations (Gimenez et al., 2018; Lebreton, 2009; Lebreton et al., 1992) but also contributed to advancing ecological theory (Cooch et al., 2002; Serrano et al., 2005). Estimating dispersal and site‐specific survival made possible through incorporating large‐scale spatial information in multi‐site (multi‐state) models (Arnason, 1972; Brownie et al., 1993). Later, spatial capture–recapture (SCR) models (Borchers & Efford, 2008; Efford, 2004; Royle & Young, 2008) unified population and landscape ecology by incorporating fine‐scale spatial information associated with individual detections into population models.

Like capture–recapture models, SCR models link individual‐level local processes to patterns at the levels of populations. Space is naturally important in capture–recapture methods as animals closer to detection devices are more likely to be detected (Box 1). This spatial information is incorporated both in the observation and ecological processes of SCR models. Using the spatial information inherent in individual detections, SCR methods eliminate the need for ad hoc estimation of the effective sampling area, hence allowing for estimation of density (Borchers & Efford, 2008). SCR methods also account for, and in fact use, spatial heterogeneity in detectability of individuals (Efford et al., 2013; Moqanaki et al., 2021). SCR models provide a species distribution model that allows a map of distribution of individual activity centers (i.e., density) to be estimated with the associated uncertainty. This allows revealing spatial variation in density with or without spatial covariates.

BOX 1 **Spatial capture–recapture (SCR) is a hierarchical capture–recapture model conditional on the distribution of animals in space.**

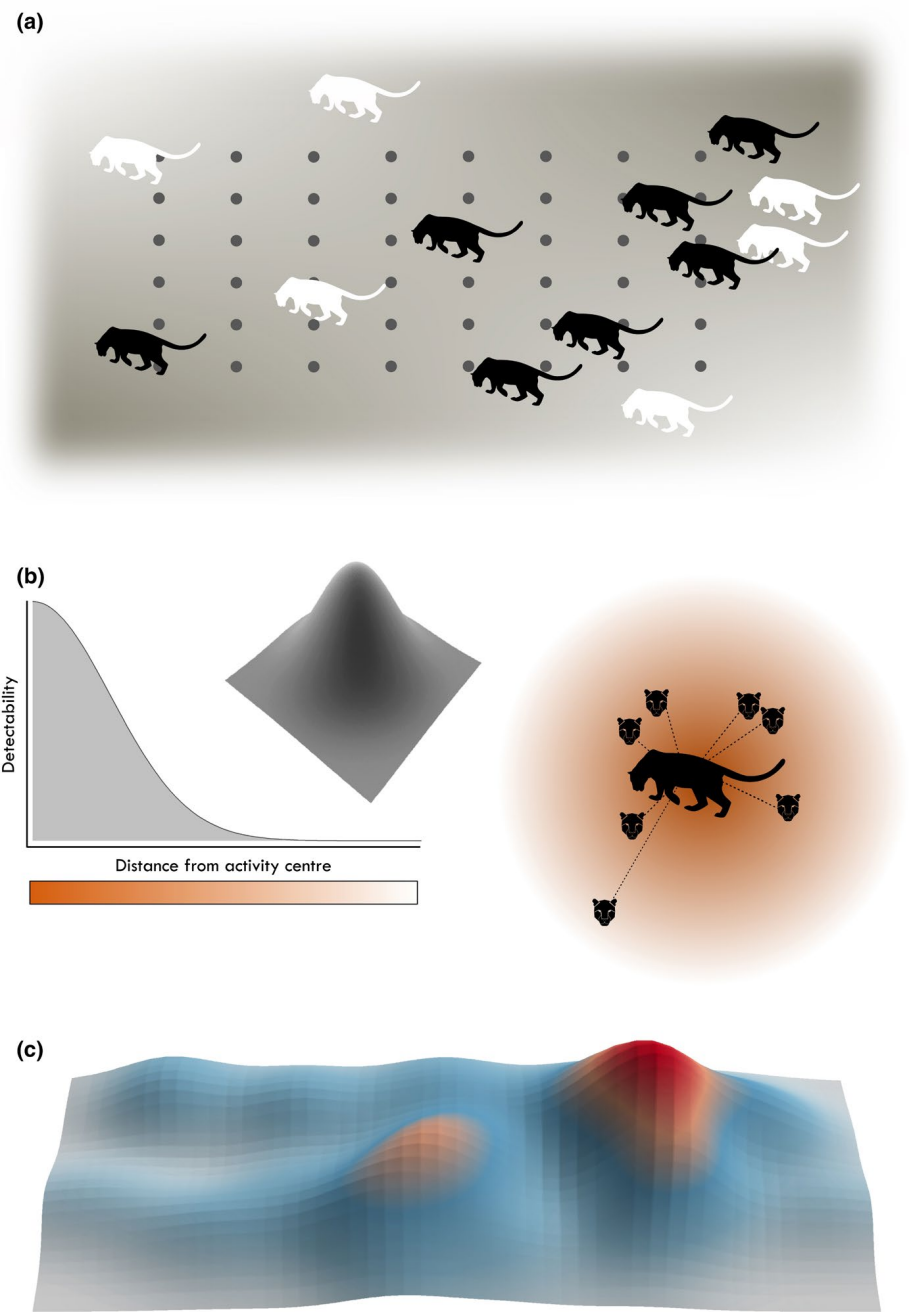

A typical SCR model consists of (i) a model of the observation process: the expected frequency (or probability) of detecting an individual at discrete locations (i.e., detectors) depends on the individual's location in space and is commonly modeled as a decreasing function of distance between the individual's center of activity and the detector; and (ii) a spatial point process model that describes how the number and distribution of animals in space arise. The goal is to draw inferences about spatial distribution of the activity centers of a population while neither their locations nor their number are observed. The main parameters estimated (or derived) are density, parameters of the detection function, and potentially covariate effects on these parameters. Figures (a–c) show a schematic description of SCR. Figure (a) illustrates a hypothetical population with individuals distributed across the space. Undetected part of the population is shown in white, while black individuals have been detected at least once at detector locations (shown as gray dots). Figure (b) shows the detectability of one individual (right panel) according to a half‐normal detection function (left panel). Animal faces indicate detection locations of the individual within its home range. Detectability is higher closer to the animal home range center. Figure (c) is an example density surface derived from SCR analysis. Color gradient in this plot indicates the highest concentration of individuals in red.

Incorporating spatial information in the SCR framework paves the way for studying spatial patterns in populations open to demographic changes and the underlying spatial processes (Royle et al., 2018). Paired with advances in noninvasive survey methods during the past decades, SCR methodology has been applied to a wide range of species and habitats, allowing for population‐level inferences with direct consequences for conservation and management (Lamb et al., 2019). Emerging technologies for collecting biological data, such as noninvasive genetic sampling, allow estimation of density by a single sampling event, which reduces the cost associated with collecting capture–recapture data (Moqanaki et al., 2018; Petit & Valiere, 2006; Solberg et al., 2006).

After more than 15 years of development since the seminal paper by Efford (2004), it is now timely to review the applications of SCR and shed some light on avenues of progress. I provide an overview of the SCR scope in terms of the ecological questions answered with this tool, taxonomic groups targeted, geographical distribution, spatio‐temporal extent of analyses, and data collection methods. In addition, I highlight approaches for analytical implementation and summarize parameters targeted by SCR studies. This review features the broad application of this analytical framework to quantify population dynamics at a scale that is relevant for conservation and management, on a variety of species, including elusive species at hard‐to‐sample areas. Finally, I discuss current limitations and future directions in using SCR methods in ecological studies.

## 2 LITERATURE SEARCH

I conducted a review of the primary literature (scientific journal articles) describing applications of SCR models. I identified published SCR studies by, first, conducting searches in the ISI Web of Science using the search terms anywhere in the document (all fields): “spatial capture recapture” OR “spatially explicit capture recapture” OR “spatially‐explicit capture‐recapture” OR “spatial capture‐recapture” OR “spatially‐explicit mark‐recapture” OR “spatial mark‐resight” OR “spatial mark‐recapture” OR “spatially explicit mark recapture” OR “spatial recapture” OR “spatially‐explicit recapture” OR “spatially‐mark recapture” OR “spatial mark resight” OR “spatial mark recapture”. I restricted the search to peer‐reviewed articles published after 2004. Second, I complemented this search result by manually searching in the bibliographies of the retrieved articles, as well as all published studies citing any of the introductory SCR papers by Efford (2004), Borchers and Efford (2008), and Royle and Young (2008) in order to identify additional articles. Whenever available, I included non‐English journal articles with English abstracts. I manually filtered the initial search results to exclude articles that only mentioned, but did not use SCR, and those of simulation‐only studies. The search was conducted on 2020‐03‐13. Initially, the search yielded 477 papers that were manually screened for removing duplicates and non‐target publications based on the criteria listed above. The final database for the synthesis contained 364 studies with ecological questions that unambiguously used SCR models to analyze empirical data (Supplementary Information 1).

I extracted 11 attributes, which are listed and described in Table 1. I extracted the required information by reading the sections in each article that contained the information referenced in Table 1 and, if needed, read the articles in full to record the required information. I did not review appendices and supplementary materials, but occasionally included them for extracting the data where applicable; for example, when the data collection methods and study area were described in the supplementary materials.

**TABLE 1:** Information extracted from 364 spatial capture–recapture (SCR) studies published between 2004 and March 2020 as retrieved in this study

Variable	Description
Species	Common and scientific names of focal species
Location	Geographic coordinates and country
Spatial extent	The spatial extent of study in km^2^ used in SCR analysis as reported in each article
Temporal extent	Duration of data collection (year)
Detector type	Data collection methods, including invasive (e.g., tagging, live‐trapping) and noninvasive (e.g., acoustic, camera trapping, fecal‐DNA sampling) methods
Focal parameters	Parameters reported in the results section of each article as the focus of the study, including abundance or density, parameters of the detection function, survival, recruitment, growth rate, movement, and coefficients of covariate effects
SCR model type	Closed‐ vs open‐population models
Analytical Implementation	i. Custom specification vs. existing specialized software packages to fit SCR models: secr, oSCR, ascr, JAGS, NIMBLE, SPACECAP, etc. ii. Whether the analyses were implemented in a Bayesian or maximum likelihood framework
Ndetected	Number of individuals detected
Population size	Point estimates (mean or median) of abundance and density (100 km^−2^)
Goodness‐of‐fit test	Whether a measure of goodness‐of‐fit has been reported in the article

I extracted the vernacular names and scientific nomenclature (highest taxonomic rank possible) of all reported focal species and identified their scientific classification from class to species using R package “taxize” and “ncbi” database (Chamberlain & Szocs, 2013). I classified the focal species into broad categories of vertebrate vs. invertebrates, and terrestrial (species living predominantly or entirely on land) vs. marine environment according to the information by the International Union for Conservation of Nature (IUCN; https://www.iucnredlist.org/). For the mammal body masses, I used PanTHERIA (Jones et al., 2009). I also identified the latest IUCN's Red List assessment in 2020 for all focal species as an index of conservation status.

For the study areas, I defined zoogeographic region according to Holt et al. (2013) and continents according to the Natural Earth (www.naturalearthdata.com). I gathered approximate geographic coordinates of the centroid of study areas, either from information provided in the paper or by searching for the study location via Google maps (https://www.google.com/maps). If the study was conducted in multiple locations within the same landscape (c. 10,000 km^2^), I chose the approximate centroid of the larger area encompassing all the study areas. The size of study area (km^2^) was taken from the article either as the reported size of state‐space (i.e., detector grid and surrounding habitat buffer) or the total area where detectors were placed.

Multiple determinations could be made for any given attribute of a study. For example, a study could be conducted in multiple study locations, target multiple species, and have more than one focal parameter. Therefore, the sample size varied based on the attribute of interest (Table 1). In cases where an article included more than one case study, I considered each case study as a separate single‐species study. I refrained from subjective classification of SCR literature into methodological and empirical studies since case studies may involve customization of the standard SCR model. I used R for tabulating the information and summaries of attributes (R Development Core Team, 2019).

## 3 PATTERNS IN SCR STUDIES

### 3.1 Parameters of interest

SCR allows capture–recapture methods to be used to address questions of a fundamentally spatial nature. Aside from providing rigorous estimates of population density (in addition to abundance), SCR methods provide a means of modeling wildlife distribution in space, as well as investigating the drivers of this distribution, drivers of habitat use, and connectivity (Royle et al., 2018).

**FIGURE 1:**
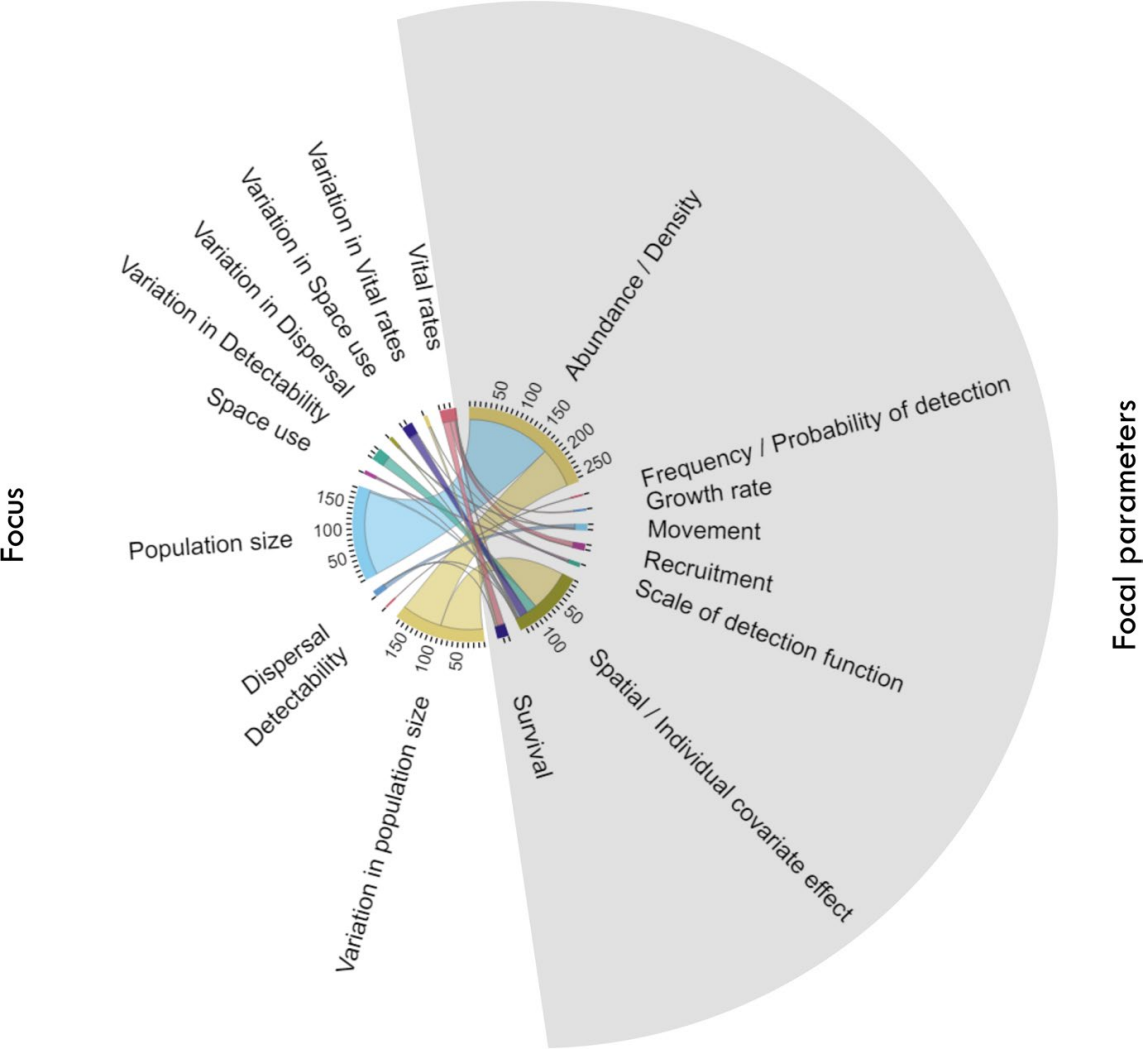
Ecological insights (left) and focal parameters (right) in published spatial capture–recapture (SCR) literature. Focal parameters of interest in SCR framework include density and abundance (closed‐ or open‐population models), and survival, recruitment, growth rate, and movement in an open‐population SCR model. Parameters of detectability depend on choice of detection model; assuming a half‐normal detection function, the spatial scale parameter of detection function may inform about space use and may be the focus of analysis besides frequency or probability of detection (the magnitude parameter). Variation in focal parameters is modeled in association with spatial, temporal, or individual level covariates in a regression formula and the effect sizes or *β* coefficients are reported as parameters of interest in analysis of covariate effects

The most common parameters estimated and reported in the reviewed articles using SCR (76%) were density, abundance, or variation associated with these measures (Figure 1). A small proportion of studies (3%; *n* = 15) reported parameters related to space use and dispersal. Frequency or probability of detection was the focus of 5% of SCR studies (*n* = 23).

Vital rates (e.g., survival, recruitment, mortality, and growth rate) and variation in these parameters were estimated in 7% (*n* = 32) of SCR studies. At least 44% of studies had data available for more than one year; however, only 7% analyzed data with open‐population SCR, that is, by explicitly modeling population dynamics.

### 3.2 Taxonomy

**FIGURE 2:**
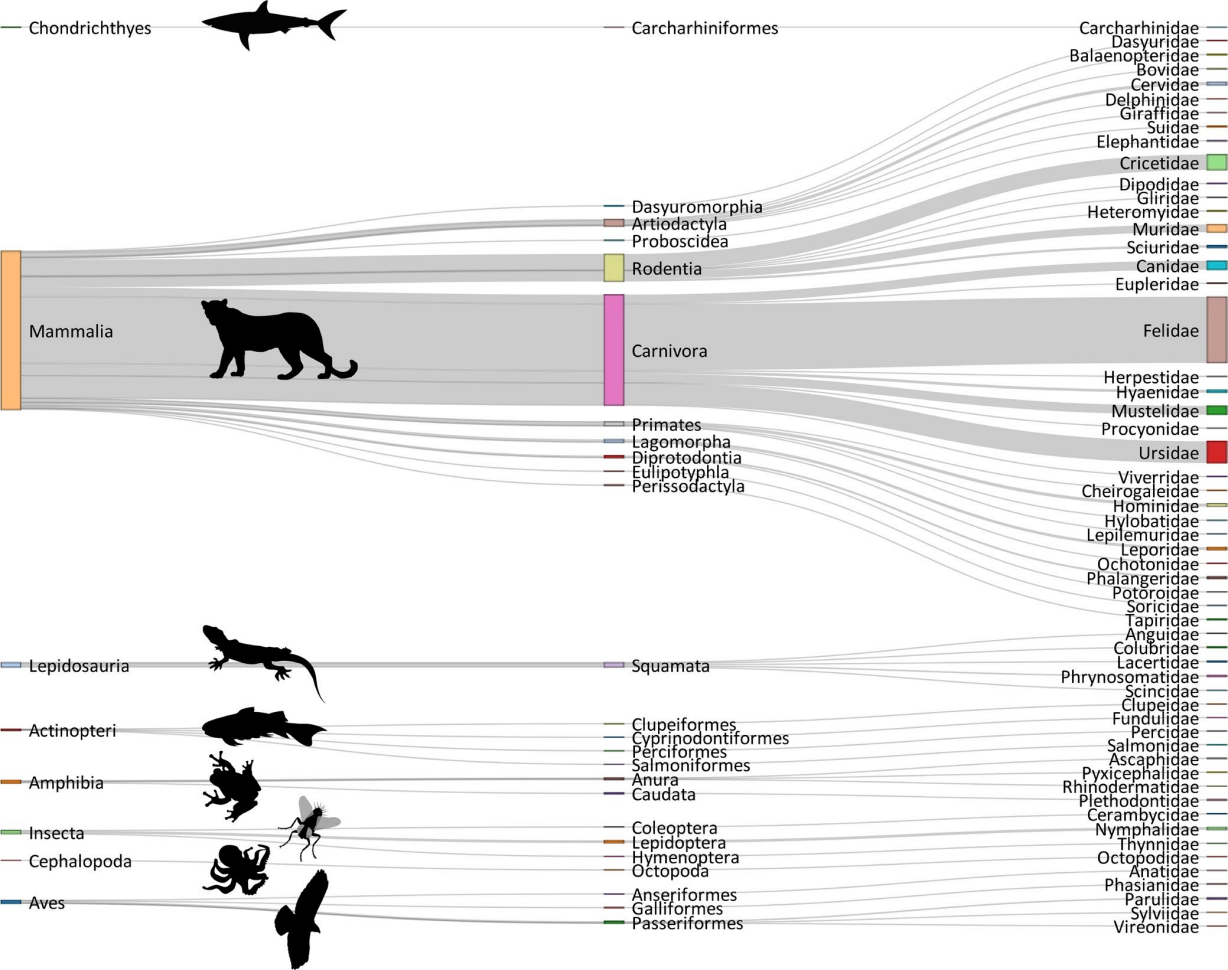
Taxonomic groups targeted in spatial capture–recapture studies according to a literature search of journal articles through ISI Web of Science in March 2020. From left to right: Class, Order, and Family based on the IUCN Red List assessments in 2020

SCR studies targeted 157 species (excluding studies that reported higher taxonomic levels) from 56 families, 25 orders, and 8 classes (Figure 2). These included a broad spectrum of species and environments, ranging from invertebrates (e.g., butterflies and common octopus *Octopus vulgaris*) to snakes (e.g., queen snake *Regina septemvittata*), marsupials (e.g., woylie *Bettongia penicillata*), sharks (e.g., gray reef shark *Carcharhinus amblyrhynchos*) and large marine mammals (e.g., Minke whale *Balaenoptera acutorostrata*). SCR studies on invertebrates were rare, currently restricted to 2% (*n* = 10) of studies. Most studies focused on mammals (*n* = 411; 90%), and within mammals, the majority of studies were on the Order Carnivora (*n* = 287; 70%), followed by Rodentia (*n* = 71; 20%), Artiodactyla (*n* = 19; 5%), and primates (*n* = 12; 3%). The species with the greatest number of SCR studies were Leopard *Panthera pardus* and tiger *P*. *tigris* (*n* = 35; 8% articles each), followed by the American black bear *Ursus americanus* (*n* = 32; 7%) and brown bear *U*. *arctos* (*n* = 21; 5%). Most reviewed studies used SCR to study a single focal species (*n* = 316; 87%), and the remaining multi‐species studies targeted between 2 and 10 focal species (median = 2). Based on the IUCN Red List assessments in 2020, 30% of the reviewed studies focused on at least one species in the threatened categories of Critically Endangered, Endangered, or Vulnerable.

### 3.3 Geographic distribution

**FIGURE 3:**
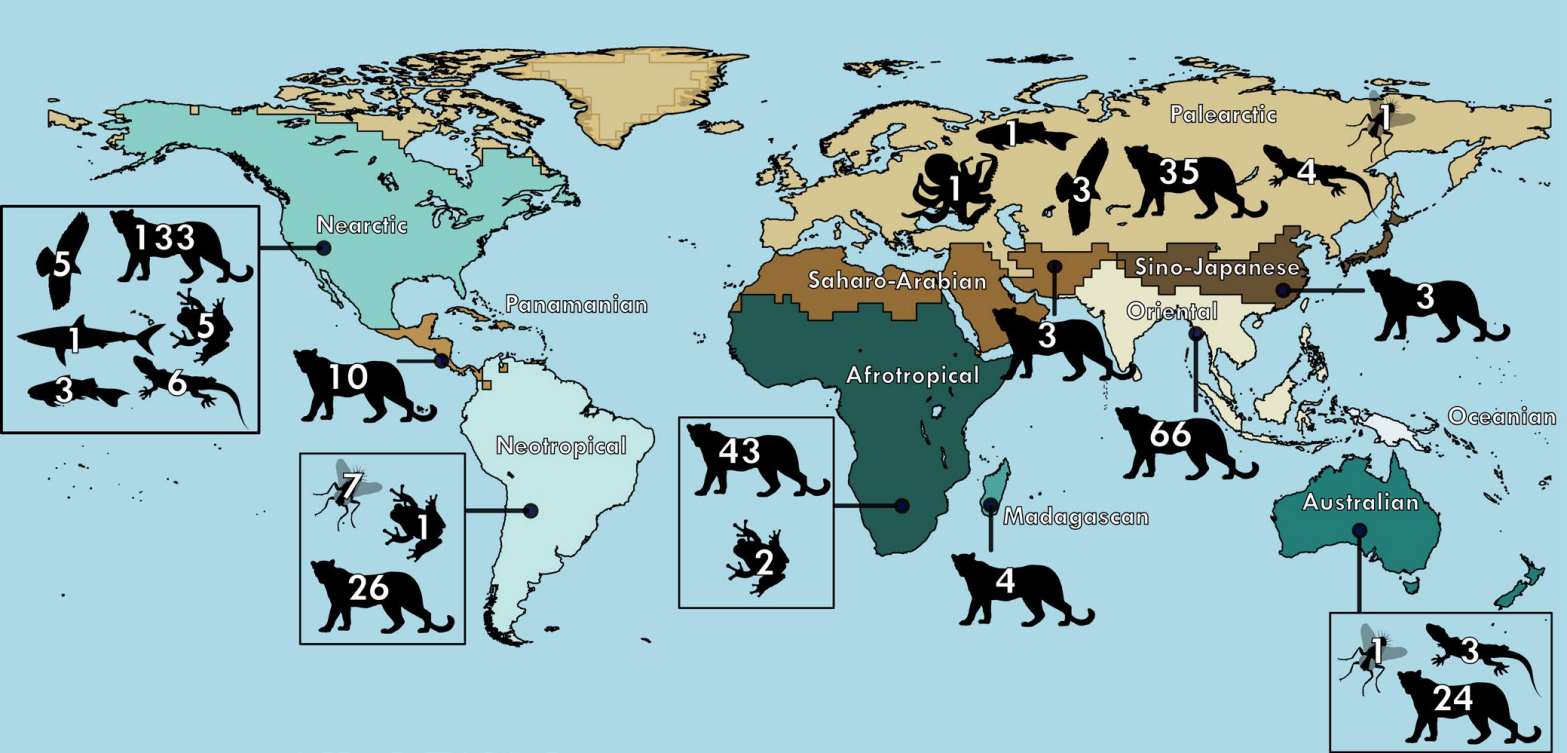
Terrestrial zoogeographic realms of the world based on Holt et al. (2013), excluding Antarctica, showing the geographic distribution of spatial capture–recapture studies by March 2020 in ISI Web of Science. Number of studies is shown per Class in each region (see the corresponding animal silhouettes in Figure 2)

SCR has found its way into studies of wildlife populations across a wide range of zoogeographic realms (Figure 3). The greatest number of studies were in the Nearctic (*n* = 156; 35%), followed by Palearctic (*n* = 86; 20%), Oriental (*n* = 67; 15%), and Afrotropical regions (*n* = 45; 10%), and SCR was less prevalent in other regions.

### 3.4 Spatial and temporal extents

**FIGURE 4:**
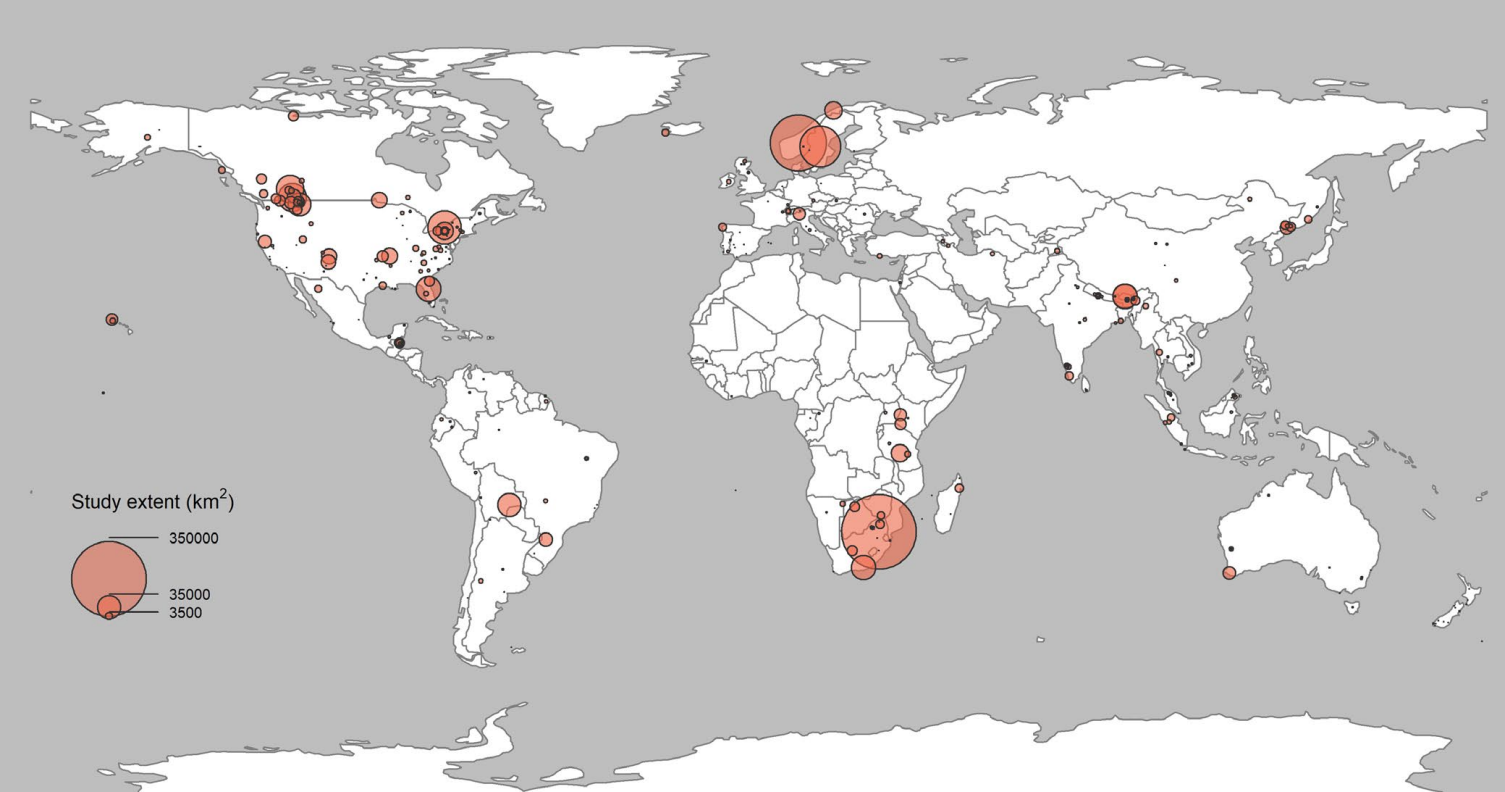
Approximate geographic distribution of published spatial capture–recapture studies by March 2020 accessed through ISI Web of Science. The size of circles indicates the relative size of study areas

SCR studies varied substantially in terms of their spatial extent, ranging from local (>1 km^2^) to landscape‐level (>10,000 km^2^) applications (Figure 4). The median of reported spatial extents was 300 km^2^ (mean = 4,077 km^2^, range: 0.0001–350,000 km^2^). However, the range sizes differed greatly depending on the focal species. Within mammals, the smallest study extents were for rodents (median = 0.5 km^2^) with median body mass of 26 g, whereas elephants have been studied across spatial extents as large as 3,000 km^2^. However, there was a great variation in spatial extent at lower taxonomic groups (Supplementary Information 2; Figure S2.1). For example, the top ten largest study extents targeted large carnivores with median body mass of 52 kg.

Landscape‐level applications of SCR, that is, study areas beyond 10,000 km^2^, were limited to 7% (*n* = 29) of the reviewed studies. Application of SCR methods involving large spatial extents (>10,000 km^2^) was limited to mammals. Overall, only 1.5% (*n* = 5) of the reviewed studies included data from transboundary (meta‐)populations (i.e., study locations in ≥2 neighboring countries).

Over 243 (56%) of the studies reviewed involved SCR data from a single year or season (Supplementary Information 2; Figure S2.2). The literature contained multiyear data for 60% of all species studied by SCR (*n* = 94), ranging from 2 to 24 years of SCR data (mean = 5, median = 3 years across all reviewed studies). The most‐studied species with multi‐year SCR data were members of class Mammalia (90%, *n* = 168). Overall, 3% (*n* = 14) of articles contained SCR data across large spatial extents for more than one year, belonging to 9 species, all mammals.

### 3.5 Data collection methods

**FIGURE 5:**
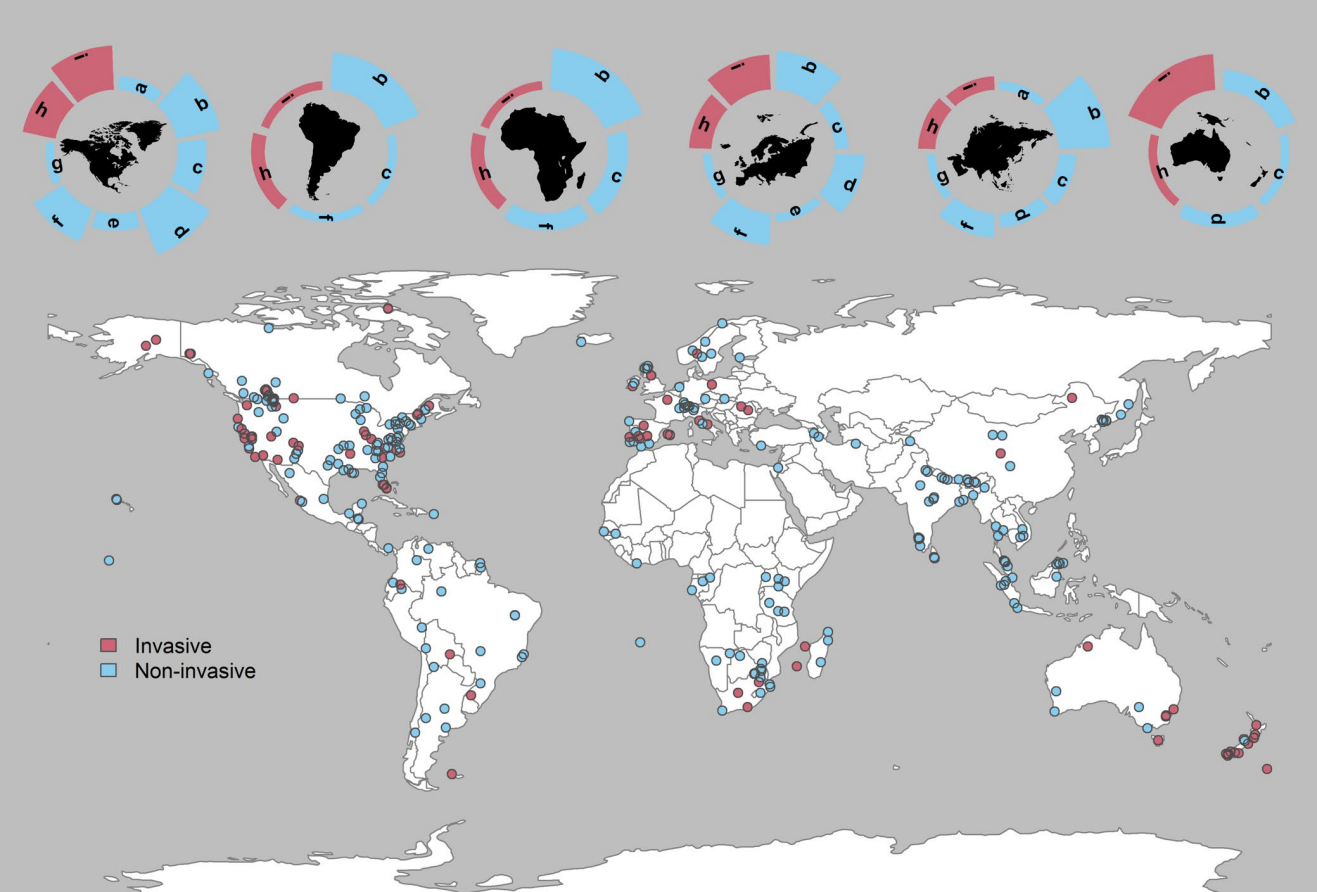
Data collection methods used in published spatial capture–recapture studies, including noninvasive (a. acoustic, b. camera trap, c. direct observation, d. DNA sampling of hair, e. other noninvasive DNA sampling, f. scat DNA, g. sign survey) and invasive sampling methods (h. telemetry and i. trapping). Log of number of studies is shown in bar graphs for each continent (top panel from left to right: North America, South America, Africa, Europe, Asia, and Oceania). Asia, Africa, and South America have the highest number of studies with noninvasive data collection methods (94%, 92%, and 88% of studies in these continents, respectively)

Available sampling techniques were broadly classified into two categories of invasive (e.g., trapping and telemetry) and noninvasive (acoustic, camera trapping, direct observation, and hair and fecal DNA sampling; Figure 5). Invasive methods are those involving physical capture and handling of study animals, whereas noninvasive methods do not require such procedures (Zemanova, 2020). Of the reviewed studies, 70% (*n* = 321) analyzed data collected noninvasively (40% camera trapping, 15% hair tagging, 10% fecal‐DNA sampling, 3% direct observations, and 2% acoustic), whereas 6% of SCR studies used a combination of more than one noninvasive method (e.g., camera trapping and DNA sampling). Six percent (*n* = 28) of studies used a combination of at least one noninvasive and one invasive sampling methods to collect SCR data, either for comparison of methods or integration of data (Figure 5). All studies of Actinopteri (*n* = 4), Cephalopoda (*n* = 1), and Insecta (*n* = 3) and most studies of Lepidosauria (90%, *n* = 14) were based on invasive sampling methods. Studies of Amphibia, Aves, and Mammalia were predominantly based on noninvasive data collection methods with 75%, 55%, and 72%, respectively.

Noninvasive DNA‐based technologies have most frequently been used in North America and northern Europe; 90% of all studies that used noninvasive genetic sampling as data collection method were conducted in these continents (*n* = 109). In contrast, camera trapping data dominated in SCR studies across Africa, Asia, and South America (75% of all studies in these continents used camera trapping; *n* = 102).

### 3.6 Analytical implementation

Parameter estimation and inference based on maximum likelihood methods was the most common approach for SCR analysis involving inference about density and abundance. Of all studies, 40% used R package “secr” (Efford, 2020), 17% “DENSITY” (Efford et al., 2004), and 3% “oSCR” (Sutherland et al., 2019). Fewer closed population studies used Bayesian methods; for example, JAGS and SPACECAP were used in 13% of all studies each (Gopalaswamy et al., 2012; Royle et al., 2014). However, all but one applications of open‐population SCR models (*n* = 31; 7% of all studies) relied on a Bayesian formulation.

### 3.7 Goodness‐of‐fit

Goodness‐of‐fit testing is a tool for comparing the observed data with the data expected under the model using some discrepancy measure, such as residuals, chi‐square, or deviance (Pradel et al., 2005). Goodness‐of‐fit testing is an important element of any analysis since it reduces the risk of drawing erroneous inference and helps diagnosing possible violation of model assumptions. Only 8% of articles (*n* = 35) reported a measure of goodness‐of‐fit test.

## 4 DISCUSSION

I have highlighted realized applications of SCR in revealing complex ecological state dynamics that are inherently difficult to observe. Availability of large datasets, noninvasive data collection technologies, and computational development have led to remarkable progress in investigating spatio‐temporal population dynamics at a scale that is relevant for conservation and management. While processes operating at fine spatial and temporal scales are likely to impact dynamics at large spatial scales, such as species' distributions, there remains a pivotal role for the SCR framework that link local (individual level) processes to large‐scale spatial population dynamics.

### 4.1 Density

Density estimates are the main goal of a wide range of studies in ecology and conservation, to make comparisons of estimates possible, for example, between different study areas and years, and results can be put into an applied context by assessing conservation status, evaluating the consequences of management interventions, or optimizing survey designs. Among reviewed articles with the focus of population size (*n* = 187), 44% (*n* = 82) modeled variability in density in response to spatial variation in one or more aspects of the environment (Figure 1). Understanding density–habitat relationships is of critical importance for predicting ecological outcomes and informing conservation actions (Hodgson et al., 2009). Knowing the factors that affect changes in the abundance of an organism can be even more critical if the species is a keystone in the community or if it is endangered and declining in numbers.

### 4.2 Detectability

Like in capture–recapture, variables related to detectability (frequency or probability of detection) may appear as nuisance parameters in SCR models but have nonetheless been the focus of only 5% of SCR studies (*n* = 23; Figure 1). Differential selection of spatial resources can be reflected in different baseline detection, and modeling effects of spatial covariates on baseline detection probability is shown as a form of resource selection modeling in SCR (Royle et al., 2013). Accounting for individual, sex‐specific, detector‐specific, and habitat type effects on detectability helps improving the estimation of other parameters, including density and space use. Behavioral response to detection devices is another ecological insight gained by modeling detectability (Romairone et al., 2018).

### 4.3 Target species

SCR studies typically use individually identified encounter history data of focal species, which, depending on species and study system, often require expensive sampling techniques and trained surveyors. Resource limitation may constrain the possibility of collaboration and exchange of skills required to analyze such data within an SCR framework. However, many species of conservation concern occur in developing regions (Jantz et al., 2015) and have been recently the focus of SCR studies to, for example, derive density estimates for conservation assessment (e.g., Gupta et al., 2019). Carnivores are the most targeted taxonomic groups in SCR studies (Figure 2) and the most studied species are charismatic species of conservation or management interest. This is in line with the global research bias toward carnivores (dos Santos et al., 2020).

### 4.4 Spatial extent

The development of noninvasive data collection methods combined with research interests in collecting data for wide‐ranging, large species, such as large carnivores (>15 kg; Ripple et al., 2014), has engendered an increase in the spatial extent of SCR studies (Figure 4). This allows researchers to make spatially explicit inferences for entire landscapes and populations (e.g., Rogan et al., 2019). In addition, SCR studies of transboundary populations allow extraction of parameters, such as abundance and density, for the constituent jurisdictions without leading to problematic double‐counting that results from isolationist approaches to monitoring (Bischof et al., 2016, 2020).

### 4.5 Data collection methods

All except one of the landscape‐scale studies (>10,000 km^2^) reviewed here (*n* = 29) used data collected by at least one noninvasive survey method, which might indicate the combined benefits of advancements in and popularity of noninvasive sampling methods that can produce large amount of data and SCR analysis (Lamb et al., 2019). Direct observation and sign surveys (e.g., spoor counts or snow tracking) are also traditionally used in vast areas in, for example, the African savanna as a low cost and minimally invasive research technique to study rare or elusive wildlife (Bauer et al., 2015). However, the contribution of these data collection methods to SCR studies I reviewed were negligible (Figure 5), possibly because they typically do not allow reliable individual identification (but see Law et al., 2013).

SCR literature across Asia, Africa, and South America mostly used camera trapping (75% of all studies in these continents). This can be in part explained by availability of resources as I outlined before, and in part due to differential suitability of selected methods in specific regions. For example, camera trapping is more common in the tropics than fecal DNA sampling, possibly as a result of the difficulty to recover usable scats of the focal species on forest floor in presence of high species misidentifications, extra costs of laboratory equipment, and technicians to collect, store, and process the samples (Abrams et al., 2019; Mondol et al., 2009). Further, genotyping success rates are generally low for biological samples collected in the presence of humidity, mold and invertebrate activities, and polymerase chain reaction (PCR) inhibitors (Beja‐Pereira et al., 2009; Vynne et al., 2012).

### 4.6 Space use

Home range information is implicitly incorporated in SCR models through a spatial detection function (Borchers & Efford, 2008). Although a small proportion of studies reported parameters related to space use (Figure 1), the observation model in SCR explicitly links the probability of detecting an individual at a given location with the distance to its latent activity center. In other words, SCR assumes a model of animal movement around a center of activity (e.g., home range center) and can yield information about space use and resource selection (Bischof et al., 2017; Efford et al., 2016; Royle et al., 2018).

### 4.7 Data integration

Noninvasive capture–recapture data typically consist of few observations of each individual to allow fitting complex detailed home range models, such as those possible based on telemetry data. The relative sparsity of data per individuals is compensated for by the number of individuals for which such data are available. However, integrating telemetry data into SCR models help fit more realistic models of home range and movement (Gardner et al., 2018; Royle et al., 2018). Integrating multiple spatial process types at different scales, including home range use and dispersal, and linking these with landscape structure can provide insights into population‐level spatial dynamics (Bischof et al., 2017; Linden et al., 2018).

### 4.8 Open‐population SCR model

The limitations of open‐population SCR models are discussed elsewhere (e.g., Gardner et al., 2018; Glennie et al., 2019). Besides model complexity and computational constrains, these models are relatively new and the paucity of open‐population SCR models for analyzing multiseason data is attributable to the fact that these models have not been around as long. Open‐population SCR models have been implemented in both Bayesian and likelihood frameworks, and there are R packages for both that are recently available (e.g., OpenCR; Efford, 2019, *OpenPopSCR*; Augustine, 2018). Open population SCR models relax some of the assumptions of closed‐population SCR models. However, these models have other set of assumptions, such as individual movement patterns, that may affect estimation of parameters (Ergon & Gardner, 2014; Efford & Schofield, 2020).

Explicit incorporation of movement of individuals into the SCR model accommodates partial availability of animals for detection, thus distinguish between emigration (temporary and permanent) and mortality (Schaub & Royle, 2014). On a similar vein, recruitment is governed by the spatio‐temporal variation in birth and survival processes (Chandler et al., 2018). However, if the dispersal distance extends outside of the spatial extent of sampling, the scale of movement is not estimated well, and the individual will appear as dead to the model (Efford & Schofield, 2020; Gardner et al., 2018). Despite this caveat, inclusion of movement parameter improves estimation of demographic parameters (Efford & Schofield, 2020).

Density dependence is an important phenomenon in population ecology, where population vital rates are linked to population density (Murray & Sandercock, 2020). When vital rates are negatively linked to density, a population fluctuates around the carrying capacity (Sibly & Hone, 2002). The study of the direction and magnitude of density dependence is thus fundamental to the understanding of processes that regulate temporal and spatial variation in population parameters. It has always been challenging to demonstrate density dependence in wild populations because of theoretical, practical, and technical problems (Lebreton, 2009). The state‐space modeling framework in SCR allows direct estimation of density and vital rates and test for dependence between them as a future direction.

### 4.9 Implementation

Free user‐friendly software packages have been available since the introduction of the method and are still the most common platforms to analyze closed‐population SCR data. Bayesian methods have been around for nearly as long (Royle & Young, 2008) but became more accessible with the advent of efficient computing platforms and flexibility in model development (e.g., Turek et al., 2021). Users face the choice between the ease of model fitting, available documentation, and computation efficiency associated with packages or software based on maximum likelihood approaches and the flexibility offered by Bayesian platforms (Kéry & Royle, 2016). There are several motivations for customizing an SCR model, for example, for analyzing data resulting from emergent technologies (e.g., Augustine et al., 2018), including random effects of sampling locations, accommodating unconventional sampling designs, or combining data from multiple sampling methods to tackle data sparsity (e.g., Tourani et al., 2020).

### 4.10 Final remarks

Despite its potential, there remain several hurdles to the implementation of SCR describing long‐term, broad‐scale ecological dynamics. Researchers must recognize the limitations of their data and how these can be leveraged by formally linking observable phenomena to the actual ecological processes of interest. SCR has shown to integrate biotic and abiotic observations at large spatio‐temporal scales to investigate complex population‐level processes. Methodological developments, such as data integration (Chandler & Clark, 2014), continuous time detection (Borchers et al., 2014), noncircular home ranges (Sutherland et al., 2015), density‐dependent home ranges (Efford et al., 2016), and passive acoustic SCR (Stevenson et al., 2021) extend our ability to incorporate complex data structures and hierarchical relationships scaled from the individual to population and species level. Availability of software packages and comprehensive support by the community of users and developers has helped researchers formulate SCR models according to their specific ecological state and observation processes of interest.

It can be challenging, however, to tailor SCR to real data, where flexible implementation is needed. Any increase in model complexity with respect to the number of ecological states or the parameters tends to exacerbate technical challenges common to hierarchical models (Auger‐Méthé et al., 2021). When working with SCR, it is thus important to be wary of these challenges and the associated risks. Application of SCR requires a conceptualization of ecological system that is amenable to the modeling framework, as well as the identification and integration of observation processes that can provide information about the underlying system. Goodness‐of‐fit testing is a useful tool helping reduce the risk of erroneous inferences that arise from violation of model assumptions (Royle et al., 2014).

About half of the studies analyzed data over more than one year. Many important questions in ecology, such as the impact of environmental change on wild populations, can only be answered with data that extend over several years (sometimes decades) and often require records of the life histories of recognizable individuals (Clutton‐Brock & Sheldon, 2010). Long‐term SCR data have the potential for investigating impacts on demography but also dynamics of the populations (Reinke et al., 2019). Individual detection histories across multiple years make it possible to go beyond estimating density for a single season and allow analyzing trends in population size and link different aspects of population demography and dynamics to space (Chandler et al., 2018). There were studies on small and isolated populations (i.e., truly population level; e.g., Cove et al., 2018), studies on small fractions of a larger population (e.g., Bradley et al., 2017), and studies on large portions of large populations (e.g., Rogan et al., 2019). Landscape‐level inferences, however, continue to be limited by compatibility and availability of data to the research community, which can compromise our ability to empirically link observation and ecological state processes operating at different spatio‐temporal scales. Using standard sampling protocols for collecting data in different projects and national or regional agreements of data sharing may facilitate landscape or regional analysis of population‐level data within an SCR framework.

## CONFLICT OF INTEREST

None declared.

## AUTHOR CONTRIBUTIONS

**Mahdieh Tourani:** Conceptualization (lead); data curation (lead); formal analysis (lead); investigation (lead); methodology (lead); validation (lead); visualization (lead); writing‐original draft (lead); writing‐review and editing (lead).

## Supporting information

Fig S1

Fig S2

Supplementary Material

## Data Availability

Articles included in the literature review are listed in the supplementary material.
