# Supplementary figures and images for: A review of spatial capture–recapture: Ecological insights, limitations, and prospects
Source: Ecol Evol. 2021 Dec 21;12(1):e8468. doi: 10.1002/ece3.8468 (PMC8794757; doi:10.1002/ece3.8468)

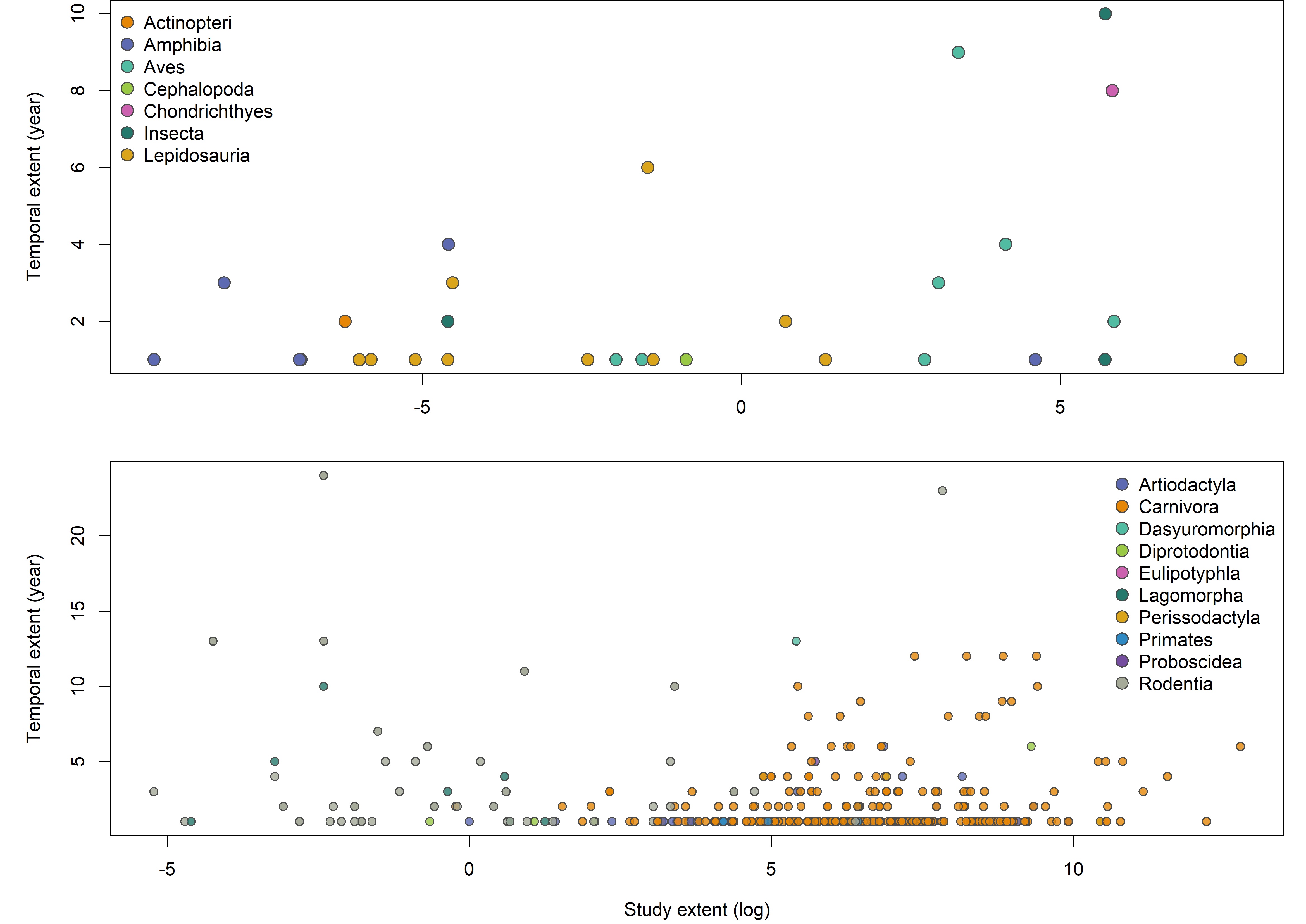

Supplement: Supplementary file 1 — Fig S1 [file ECE3-12-e8468-s001.jpg]

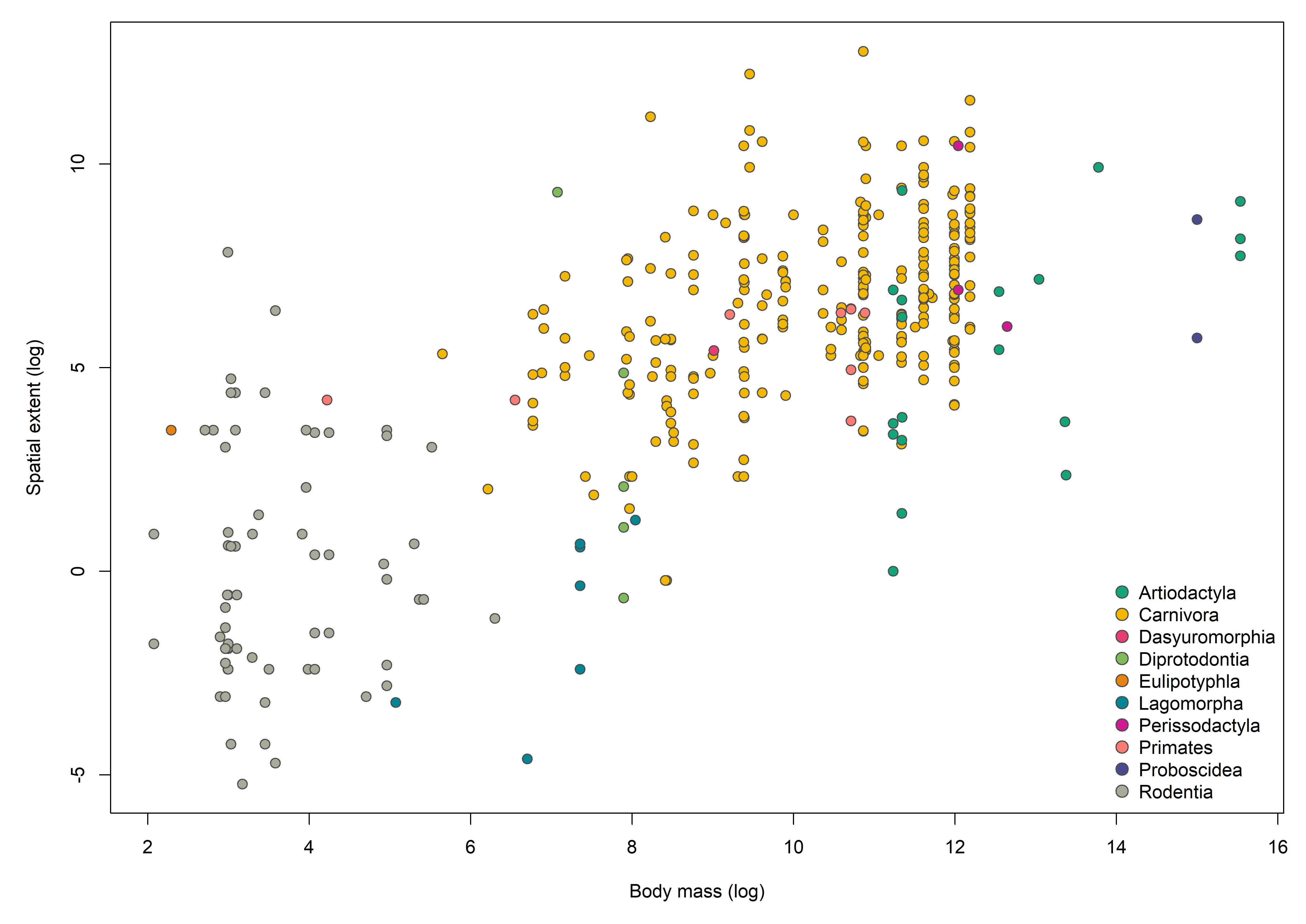

Supplement: Supplementary file 2 — Fig S2 [file ECE3-12-e8468-s003.jpg]
